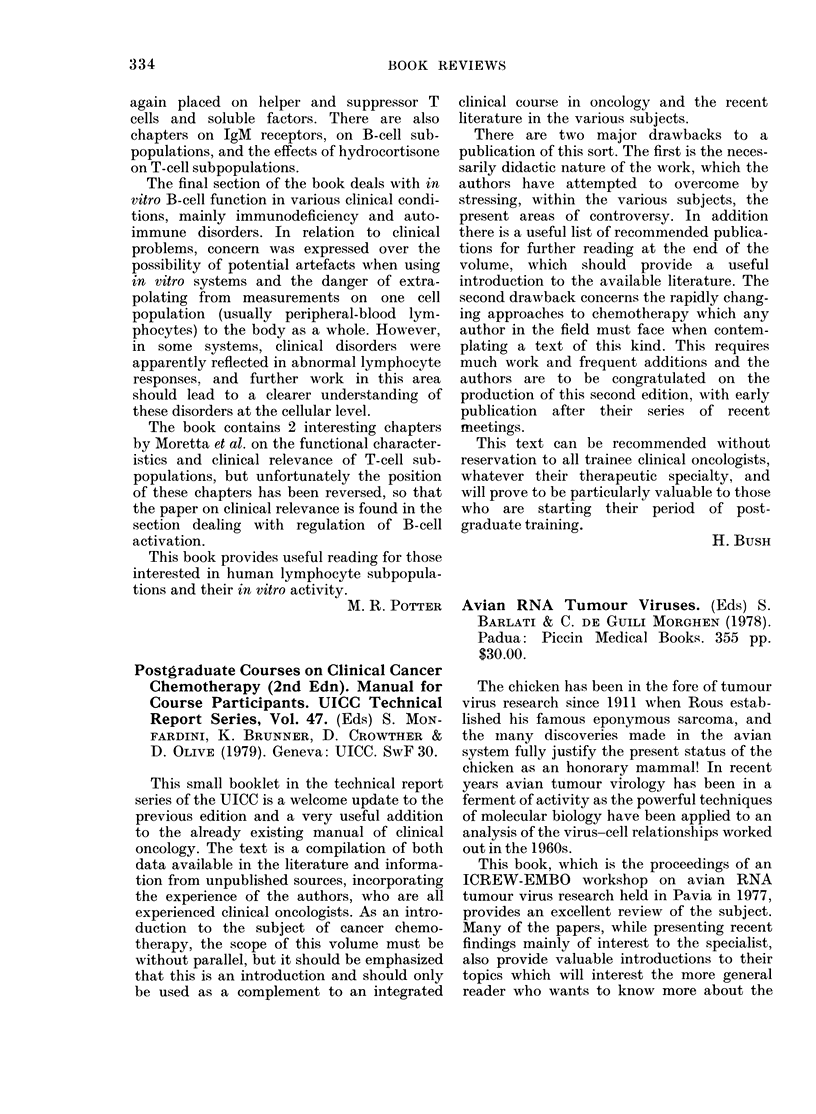# Postgraduate Courses on Clinical Cancer Chemotherapy (2nd Edn). Manual for Course Participants. UICC Technical Report Series, Vol. 47

**Published:** 1980-02

**Authors:** H. Bush


**Postgraduate Courses on Clinical Cancer Chemotherapy (2nd Edn). Manual for Course Participants. UICC Technical Report Series, Vol. 47.** (Eds) S. MONFARDINI, K. BRUNNER, D. CROWTHER & D. OLIVE (1979). Geneva: UICC. SwF 30.

This small booklet in the technical report series of the UICC is a welcome update to the previous edition and a very useful addition to the already existing manual of clinical oncology. The text is a compilation of both data available in the literature and information from unpublished sources, incorporating the experience of the authors, who are all experienced clinical oncologists. As an introduction to the subject of cancer chemotherapy, the scope of this volume must be without parallel, but it should be emphasized that this is an introduction and should only be used as a complement to an integrated clinical course in oncology and the recent literature in the various subjects.

There are two major drawbacks to a publication of this sort. The first is the necessarily didactic nature of the work, which the authors have attempted to overcome by stressing, within the various subjects, the present areas of controversy. In addition there is a useful list of recommended publications for further reading at the end of the volume, which should provide a useful introduction to the available literature. The second drawback concerns the rapidly changing approaches to chemotherapy which any author in the field must face when contemplating a text of this kind. This requires much work and frequent additions and the authors are to be congratulated on the production of this second edition, with early publication after their series of recent meetings.

This text can be recommended without reservation to all trainee clinical oncologists, whatever their therapeutic specialty, and will prove to be particularly valuable to those who are starting their period of postgraduate training.

H. BUSH